# The role of *Proteus mirabilis* cell wall features in biofilm formation

**DOI:** 10.1007/s00203-016-1249-x

**Published:** 2016-06-04

**Authors:** Grzegorz Czerwonka, Anna Guzy, Klaudia Kałuża, Michalina Grosicka, Magdalena Dańczuk, Łukasz Lechowicz, Dawid Gmiter, Paweł Kowalczyk, Wiesław Kaca

**Affiliations:** 1 Department of Microbiology, Jan Kochanowski University, Świętokrzyska 15, 25-406 Kielce, Poland; 2 Department of Biobanking and Scientific Research, The Regional Science and Technology Center, Podzamcze 45, 26-060 Chęciny, Poland; 3 Faculty of Environmental, Geomatic and Energy Engineering, Kielce University of Technology, al. Tysiąclecia Państwa Polskiego 7, 25-314 Kielce, Poland; 4 Bionicum LTD, Chełmska 21, 00-724 Warsaw, Poland

**Keywords:** *Proteus mirabilis*, Biofilm, Cell surface hydrophobicity (CSH), Microbial adherence to hydrocarbons (MATH), Electrokinetic potential, Epifluorescence microscopy

## Abstract

Biofilms formed by *Proteus mirabilis* strains are a serious medical problem, especially in the case of urinary tract infections. Early stages of biofilm formation, such as reversible and irreversible adhesion, are essential for bacteria to form biofilm and avoid eradication by antibiotic therapy. Adhesion to solid surfaces is a complex process where numerous factors play a role, where hydrophobic and electrostatic interactions with solid surface seem to be substantial. Cell surface hydrophobicity and electrokinetic potential of bacterial cells depend on their surface composition and structure, where lipopolysaccharide, in Gram-negative bacteria, is prevailing. Our studies focused on clinical and laboratory *P. mirabilis* strains, where laboratory strains have determined LPS structures. Adherence and biofilm formation tests revealed significant differences between strains adhered in early stages of biofilm formation. Amounts of formed biofilm were expressed by the absorption of crystal violet. Higher biofilm amounts were formed by the strains with more negative values of zeta potential. In contrast, high cell surface hydrophobicity correlated with low biofilm amount.

## Introduction

*Proteus mirabilis* is one of the causes of catheter-associated urinary tract infections (CAUTIs). Ability to grow in the form of biofilm seems to be one of the most important virulence factors of *P. mirabilis* (Jacobsen et al. 2008). Biofilm is a complex structure formed by a single- or multi-species community of bacteria. Microorganisms in biofilm are surrounded by a self-produced extracellular polymeric substance (EPS). Five steps of biofilm formation are mentioned in literature, following: (1) reversible and (2) irreversible adhesion, (3) microcolony formation, (4) maturation of the biofilm, and (5) dispersion of the bacteria (Myszka and Czaczyk 2011). Biofilm formation is one of the important pathogenic factors of Gram-negative bacteria. The first step, change of planktonic swimming cells to settled, adhered to solid surface, is crucial for biofilm formation. Unique environmental conditions in the biofilm matrix provide protection against antimicrobial agents and prevent eradication. The mechanism of this protection activity could be based on the properties of EPS, which hinder antibiotics diffusion, and changes in microorganisms’ metabolism and phenotype are also observed (Stewart 2002; Czerwonka et al. 2014). Microbial adhesion to solid surface plays a key role in biofilm formation. This process is mediated by bacterial cell wall surface structures like: lipopolysaccharide, pili, autotransporter adhesin, and other unique proteins (Hori and Matsumoto 2010). Cell surface hydrophobicity (CSH) participation in adhesion can be explained by the classical Derjaguin–Landau–Verwey–Overbeek (DLVO) theory of colloid stability (Hermansson 1999; Czaczyk et al. 2008). According to the DLVO theory, the total energy of adhesion is the result of the van der Waals attractive forces and the generally repulsive interactions due to the interpretation of the electrical double layers (Azeredo et al. 1999). Experiments on bacterial adhesion to sulphated polystyrene (hydrophobic, negatively charged) revealed that this process can be described by a combined effect of hydrophobic and electrostatic interaction (van Loosdrecht et al. 1987), whereas electrokinetic potential of cell surface seems to be more important in the first steps of adhesion than hydrophobicity. Zeta potential which is the electrical potential of the interface between the aqueous solution and the stationary layer of such a fluid attached to the bacterial cell. Cell surface hydrophobicity and cell surface charge have been found to vary due to biological activity such as varying growth of cellular metabolism and genetic differences (Kłodzińska et al. 2010). The aims of the present studies were to correlate cell electrokinetic potential, cell surface hydrophobicity, O-polysaccharide structure with abilities for biofilm formation of the laboratory strains of *P. mirabilis* belonging to O3, O17 and O18 serogroup, as well as three UTI clinical strains. The cell wall properties of swimming, planktonic cells of three laboratory strains with defined O-polysaccharide structures of LPS and three clinical strains were tested, and their influence on adhesion and biofilm formation were determined.

## Materials and methods

### Bacterial strains and growth measurement

*Proteus mirabilis* laboratory strains (PrK 61/57—O17 and PrK 34/57—O18) were obtained from the Czech National Collection of Type Cultures in Prague, Czech Republic, strains S1959—O3 were from the Institute of Microbiology, Biotechnology and Immunology of University of Łódź, and clinical strains were obtained from Holycross Cancer Center, Poland (Table 1). Strains were stored in LB broth supplemented with 8 % DMSO stock at the temperature of −80 °C (Coenye et al. 2001). Growth was examined on Infinite M200PRO microplate reader (Tecan) in 96-well flat-bottomed, transparent plates (Corning) in 200 µl LB broth inoculated with overnight culture diluted in ratio 1:100. Liquid culture was monitored for 3 h at 37 °C, and turbidity measurements were taken every 1 h after shaking the microplates for 10 s. Measurement was performed in triplicate.

**Table 1:**
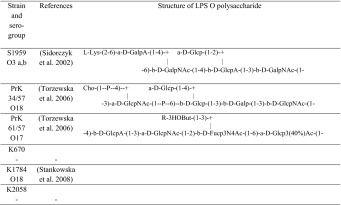
*Proteus mirabilis* carbohydrate structures

### Microbial adherence to hydrocarbons (MATH)

MATH methods were employed to determine cell surface hydrophobicity (CSH) as described earlier (Tendolkar et al. 2004), with slight modifications. Diluted bacterial strains suspension was cultivated by shaking in 50 ml probes at 37 °C for 16 h. The inoculum was centrifuged for 10 min (5000 rpm). Supernatant was discarded and pellet was resuspended in PUM buffer (22.2 g of potassium phosphate trihydrate, 7.26 g of monobasic potassium phosphate, 1.8 g of urea, and 0.2 g of magnesium sulfate heptahydrate/liter; pH 7.1) to obtain OD_600_ equal to 0.5. Suspension of bacteria was transferred into test tubes. It was mixed following the ratio of bacterial suspension to *p*-xylene (2 min; 2000 rpm): 1:0.25 and 1:0.5. Probes were incubated at RT for 30 min. After phase separation, 400 μl of water phase was gently transferred into 96-well microtiter plates and OD_600_ assay was performed. To determine CSH (%), the following formula was used: CSH (%) = [OD_600i_ − OD_600n_] OD_600i_^−1^ 100 %, where OD_600i_ is the absorbance of the initial solution and OD_600n_ is the absorbance of the solution after being mixed with *p*-xylene. According to Jones (Jones et al. 1996), bacteria with CSH (%) lower than 30 % are highly hydrophilic and with CSH (%) higher than 70 % are highly hydrophobic.

### Electrokinetic measurement

A liquid LB medium (Biocorp) was inoculated with bacterial strains and incubated for 24 h at 37 °C with continuous shaking (160 rpm). Grown cultures were washed with phosphate saline buffer PBS (pH 7.4) in triplicate to discard medium residues. Washed cells were resuspended in PBS to obtain density equal 0.5 McFarland standard. Zetasizer Nano ZS device equipped with DTS1070 cuvettes (Malvern Instrument, Malvern, UK) was used for measuring the zeta potential (ζ), which is determined from electrophoretic mobility (μ) based on Smoluchowski’s formula.

### Adherence of *Proteus mirabilis* cells to glass and polypropylene

In order to perform an assessment of the kinetics of bacterial adherence to the hydrophobic (polypropylene) and hydrophilic (glass) surface, six overnight cultures of selected strains were performed. Glass microscope slides (soda-lime glass) and polypropylene microtiter plate covers (Nunc) were used. A liquid medium LB was inoculated with bacterial strains and incubated for 24 h at 37 °C with continuous shaking (160 rpm). Cultures were diluted with liquid medium LB at ratio 1:100. Hydrophilic (glass) and hydrophobic (polypropylene) plates were placed in a vessel with bacterial culture. The cultures were incubated at 37 °C without shaking, and after every hour of incubation adherence of bacterial cells was determined with epifluorescence microscope (Carl Zeiss Axio Scope.A1). Bacteria were stained with Syto9 and propidium iodide (FilmTracer™, Invitrogen). Staining was performed according to the manufacturer’s instructions. Contact angle measurements of glass and polypropylene were performed in the Institute of Physics of the Jan Kochanowski University.

### *Proteus mirabilis* biofilm determination

Biofilm formation was performed in LB medium (Biocorp), Christensen broth (Cullen et al. 2015) and in Artificial Urine (Stankowska et al. 2012) at 37 °C in 96-well microtiter plates (Nunclon). Overnight culture was diluted (1:100) and transferred into wells in volume 200 μl and incubated overnight at 37 °C with a cover. The broth was removed, biofilm was triple-washed with 0.9 % NaCl solution (200 μl). The 200 μl of 0.01 % (w/v) crystal violet solution was added, incubated at room temperature (RT) for 15 min. and washed five times with 0.9 % NaCl solution. The washed wells were filled with 200 μl of 95 % ethanol at RT for 15 min. Crystal violet absorbance was measured at the *λ* = 595 nm with an Infinite M200PRO microplate reader (Tecan). Assay was performed in at least 3 independent repeats. An average value of the absorbance was presented on the graph with standard deviation (SD) as error bars.

### Mathematical analysis

For the mathematical analysis, the following methodology was used. In the first step, the variable values were rescaled to the range 0–1 (the maximum value of the variable = 1, the minimum value of the variable = 0). Then, using the average linkage method and Chebyshev distance, dendrograms were drawn. The color scale established as follows: the lowest value {0} assigned to red and the highest value {1} to green color.

## Results

### Adherence of *Proteus mirabilis* cells to hydrocarbons—MATH

**Fig. 1:**
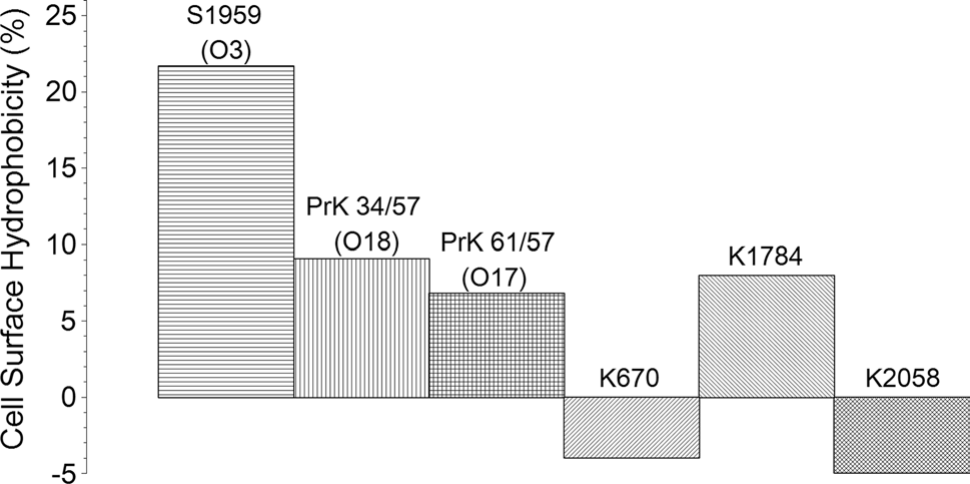
Values of the CSH (%) of *Proteus mirabilis* strains. Bacterial suspension-to*-p*-xylene ratio equal to 1:0.25

In order to exanimate cell surface hydrophobicity of the tested bacteria, the MATH method was conducted. Cell surface hydrophobicity (CSH) is expressed as a percent of the cells excluded from the water phase (Fig. 1).

Tested strains differ in CSH value, but all strains were determined as hydrophilic, according to (Jones et al. 1996). In two cases (K670 and K2058), toxicity of xylene on bacterial cells resulted in negative values. The highest value of CSH revealed in laboratory strain S1959 (22 %) was two times higher than other strains.

### Electrokinetic potential of *Proteus mirabilis* cell surface

**Fig. 2:**
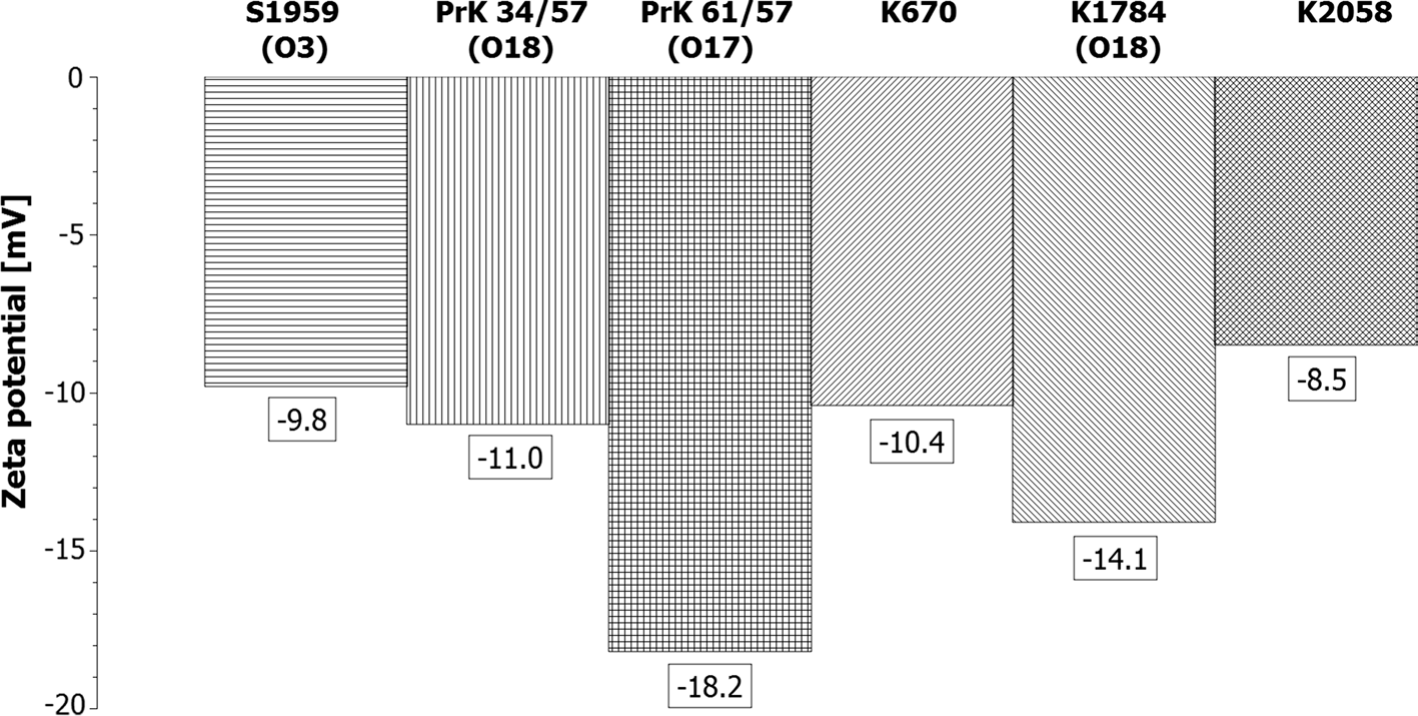
Values of zeta potential of selected *Proteus mirabilis* strains

To determine how zeta potential of cell surface is connected with O-antigen structure and how it affects adherence and biofilm formation, the zeta potential of *P. mirabilis* clinical and laboratory planktonic strains was studied. The charge of cell surface is expressed as zeta potential, and all tested strains were negatively charged (Fig. 2).

In the laboratory strain group of *P. mirabilis* strain, PrK 61/57 (O17) revealed the lowest (−18.2 mV) zeta potential and S1959 (O3) the highest (−9.8 mV). In the clinical group, K1784 (O18) revealed the lowest (−14.1 mV) and K2059 the highest (−8.5 mV) zeta potential. Strains K1784 (O18) and PrK 34/57 (O18) represent O18 serogroup with identical O-polysaccharides LPSs, and their zeta potential significantly differs (3.1 mV).

### Adherence of *Proteus mirabilis* cells to glass and polypropylene

**Fig. 3:**
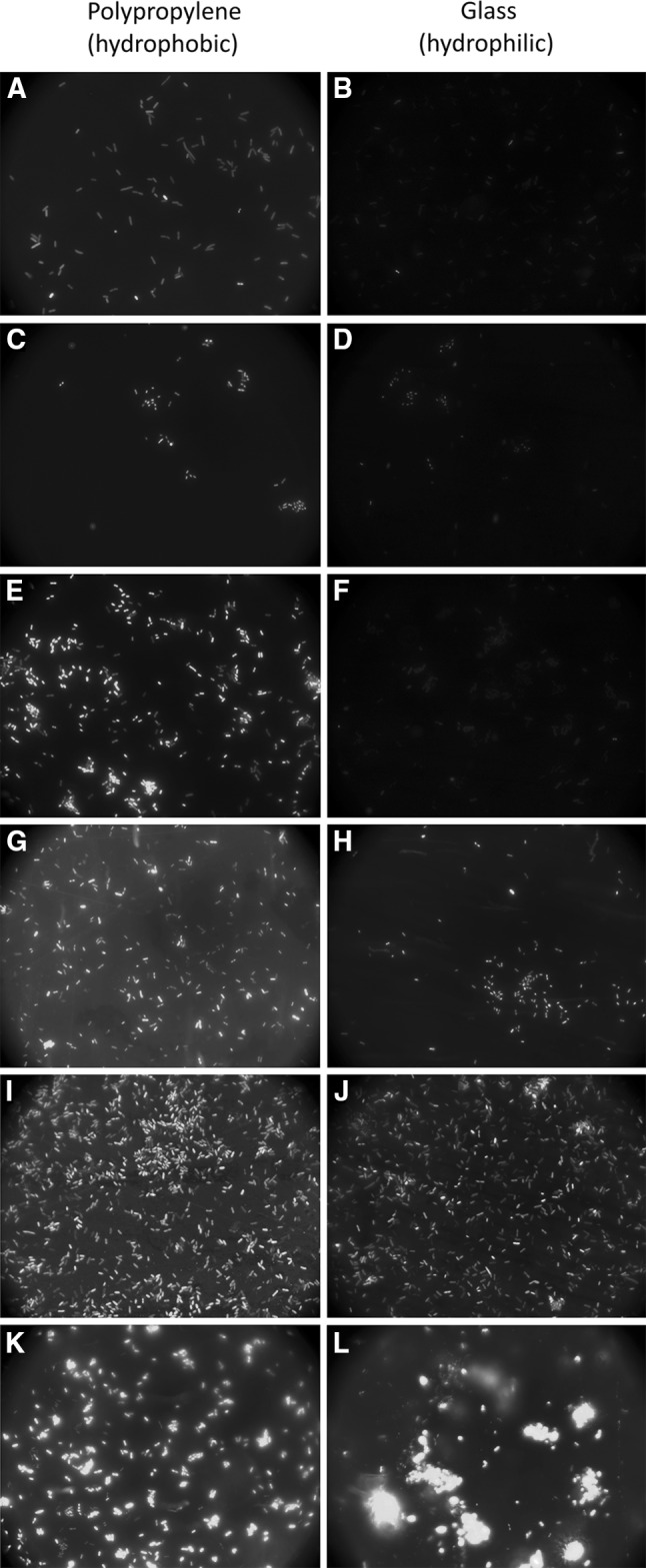
Adherence to hydrophobic and hydrophilic surface of selected strains of *Proteus mirabilis* in second hour of incubation at 37 °C. **a**, **b** S1959 (O3); **c**, **d**—PrK 34/57 (O18); **e**, **f** PrK 61/57 (O17); **g**, **h** K670; **i**, **j** K1784 (O18); **k**, **l** K2058. Representative images are presented

In order to find out whether swimming, planktonic cells are able to adhere to solid surfaces, two types of plates were used. Contact angle measurements revealed significant differences between glass plates (27°) and polypropylene (67°) which allowed to consider glass plate as hydrophilic surface and polypropylene as hydrophobic surface, and both as wettable surface (<90°) (Sobczak et al. 2004). Adherence of bacterial cells to glass (hydrophilic) and polypropylene (hydrophobic) plates revealed significant differences between the tested strains. This feature seems to be strain-specific (Fig. 3). Adherence tests were performed also in the first and third hour of incubation at 37 °C. No time dependence differences were observed. Representative pictures after the second hour of incubation were presented (Fig. 3). Much fewer cells were adsorbed on hydrophilic surface (glass) with exception to K1784 (O18) where adhesion to both surfaces (hydrophobic and hydrophilic) was similar. Other O18 strain (PrK 34/57) revealed the weakest adhesion to both surfaces in this test.

### Clinical and laboratory strains biofilm determination

**Fig. 4:**
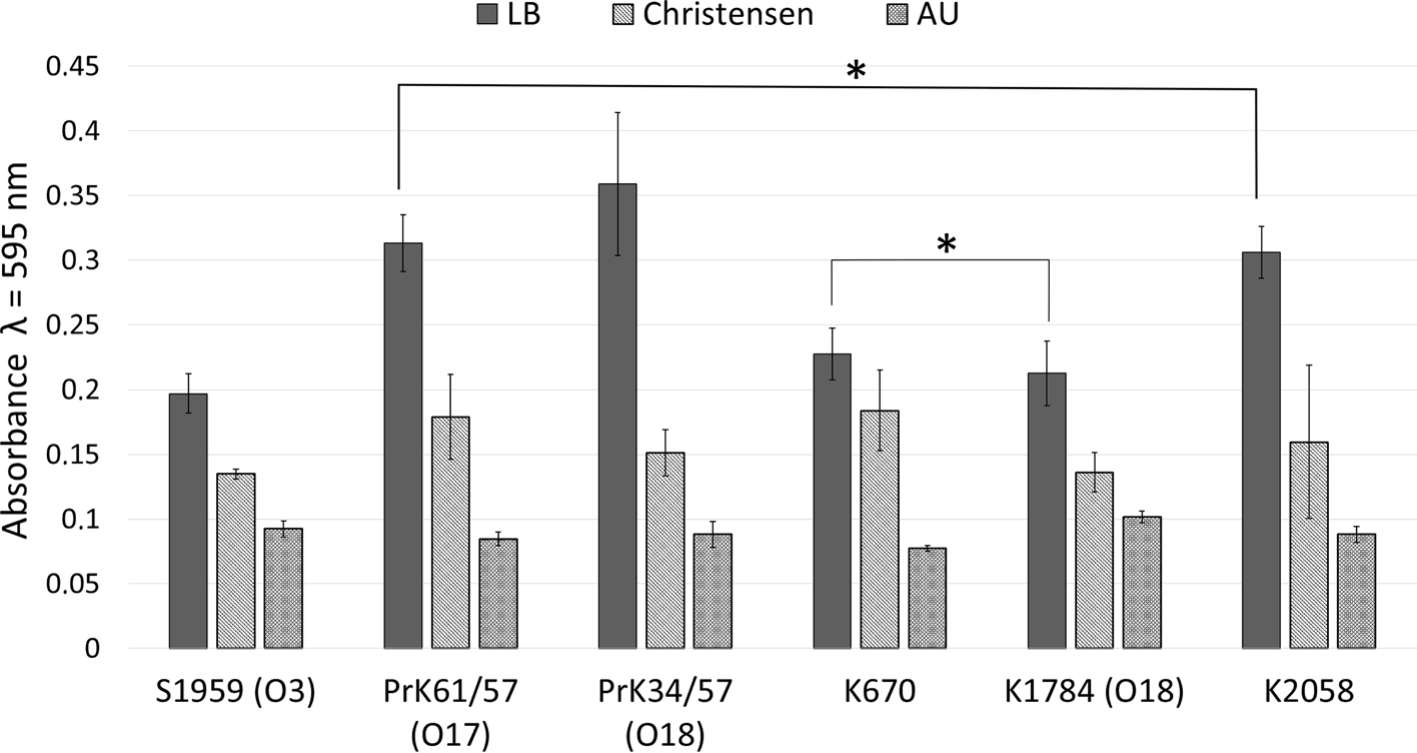
Biofilm formation by clinical and laboratory strains of *Proteus mirabilis* expressed as the absorbance of crystal violet at *λ* = 595 nm. Standard deviation is presented as *error bars*. One-way ANOVA test showed significant differences (*p* < 0.05) in presented results with exception of two cases denoted with *horizontal line* and *asterisk mark*

The ability of biofilm formation was tested on three different media: LB, Christensen, and artificial urine (AU). Adherence of the tested strains is the first step of the biofilm formation process. Strains were subjected to biofilm amount measurement, expressed as the absorbance of crystal violet at *λ* = 595 nm eluted from samples in microtiter assay (O’Toole 2011). Assays with artificial urine (AU) and Christensen broth did not reveal significant differences between strains (Fig. 4). Strain-specific differences of biofilm formation were observed on rich LB medium, and those results were subject to considerations. Laboratory strains PrK 61/57 and PrK 34/57 were classified as producers of high amount of biofilm (ABS = 0.313 ± 0.022 and 0.359 ± 0.055, respectively), while S1959 as producers of low amount of biofilm (ABS = 0.196 ± 0.015). In clinical strains, the differences were smaller, where K2058 (ABS = 0.306 ± 0.020) strains produced a higher amount of biofilm than K1784 (ABS = 0.213 ± 0.025) and K670 (ABS = 0.227 ± 0.020).

## Discussion

Adhesion of microorganisms to biotic and abiotic surfaces is a first step of biofilm formation. Cell surface hydrophobicity and cell surface charge has been considered as important factors that affect this complicated process. Three laboratory strains have determined their O-polysaccharide structure. Serogroup O3 possesses carboxyl groups, and the general charge of O-polysaccharide is negative. The charge of O-polysaccharide in serogroups O17 and O18 is close to neutral. Bacterial LPSs cover up 70 % of cell surface (Lodowska et al. 2007), so its charge may influence the total charge of cell surface and their amphiphilic nature also determines cell surface hydrophobicity. The results obtained suggest that strains with the highest CSH values (S1959) produced low amounts of biofilm expressed as amounts CV incorporated in its structure. Strain S1959 revealed the highest result in the MATH test which suggests that this strain has less hydrophilic cell surface of all the tested strains, and in the adherence assay the number of cells attached to the hydrophobic surface was higher than to hydrophilic (Fig. 1). Strains with negative values of CSH were previously considered as hydrophilic (Kotiranta et al. 1998); however, such a result could be obtained due to the toxicity of *p*-xylene. The aggressive nature of solvents could cause degradation of the cells (Czaczyk et al. 2008). In other strains with a lower CSH result, such a correlation did not occur. However, both strains of O18 serogroup PrK34/57 and K1784 possess a very similar CSH value (9 % and 8 %, respectively). This observation suggests that cell surface hydrophobicity may be connected with O-antigen structure.

**Fig. 5:**
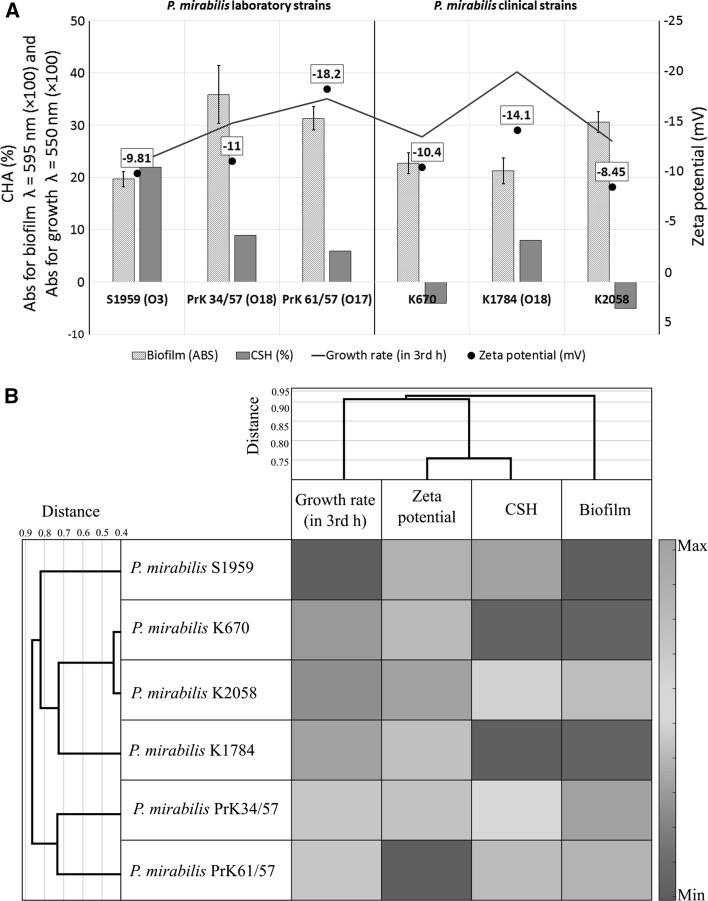
Comparison (**a**) and heatmap (**b**) of selected features: biofilm amounts (ABS), CSH (%) zeta potential (mV) and growth rate (ABS) of clinical and laboratory strains of *Proteus mirabilis*. Biofilm values and growth ratio were multiplied by 100 in order to facilitate reading of the graph

Presented data show that in the case of *Proteus mirabilis* strains, zeta potential correlates with cell surface hydrophobicity. Biofilm measured using the CV staining method seems to be correlated with growth rate rather than with zeta potential or cell surface hydrophobicity (Fig. 5b). These findings could be confirmed also by adherence tests where in the second and third hour of incubation the number of adhered cells corresponded to their growth rate (Fig. 3). Studies with a multivariate approach to investigate the influence of different factors on biofilm formation by *Pseudomonas aeruginosa*, Ruhal et al. suggest that the most important variables correlated positively with biofilm formation are hydrophobicity followed by motility (Ruhal et al. 2015). These variables were more important than zeta potential and chemical composition of the surface (Ruhal et al. 2015). On the other hand, studies on *Bradyrhizobium japonicum* lipopolysaccharide-deficient mutant indicate that O-antigen structure plays an important role in cell surface hydrophobicity (Park and So 2000). The results suggest reverse correlation between cell surface hydrophobicity and biofilm formation. In the work of Auger et al. (Auger et al. 2009), similar results were observed for *Bacillus thuringiensis* and *B. cereus* strains. *Bacillus thuringiensis* strains, in which more than 40 % of the strains formed biofilm (in microtiter PVC plates), possess more hydrophilic cells. In contrast, hydrophobic *B. cereus* strains were the weakest biofilm producers. Additionally, CSH of the *Bacillus* sp. strains partially correlated with the ability of S-layer production. Other studies focused on the role of the cell surface hydrophobicity and motility on adherence and biofilm formation on polystyrene by *Stenotrophomonas maltophilia*. The positive correlation between CSH and adherence, as well as biofilm formation, was found (Pompilio et al. 2008).

In the presented studies, higher biofilm amounts were formed by the strains with the more negative values of zeta potential, where *P. mirabilis* PrK 61/57, which possess the most negative cells surface charge (−18.2 mV), formed biofilm at a high level and less negative strains (S1959 formed lower amounts of biofilm) (Fig. 5a, b). Interestingly, adherence studies show that electrokinetic potential seems not to have any influence on adhesion to hydrophobic or hydrophilic surfaces. In general, in the results obtained, strains with a more negative cells surface charge attached to hydrocarbons with lower efficiency (Fig. 5). It is contrary with the results of Vanhaecke et al. (1990) experiment, where *P. aeruginosa* strains with a high hexadecane-partitioning value in MATH tended to have a high negative charge. Comparing all the collected data (Fig. 5), correlation between zeta potential and growth rate is visible, where highly negative charged cells seem to grow faster than cells with low charge. *Proteus mirabilis* strains demonstrated the ability to form biofilm at a stable level. This feature was observed as strain-dependent.
